# Building Wealth, Building Health: Asset Holding and Health Care Utilization Among U.S. Immigrants

**DOI:** 10.3390/healthcare13020101

**Published:** 2025-01-07

**Authors:** Haenim Lee

**Affiliations:** Department of Social Welfare and Counseling, Dongguk University, Seoul 04620, Republic of Korea; hnl22@dgu.ac.kr

**Keywords:** immigrant health, healthcare utilization, the Andersen Behavioral Model, asset holding

## Abstract

Purpose: Immigrants’ low socioeconomic status is consistently linked to reduced healthcare utilization. This study extends the Andersen Behavioral Model by incorporating asset ownership as an enabling factor and examining its role across immigrant origin groups. It addresses two questions: (1) What is the relationship between asset ownership and healthcare utilization among immigrants? (2) Are there ethnic differences in this relationship? Methods: Data from 4730 adults in the National Immigrant Survey (NIS) were analyzed. Missing income values were handled using multiple imputations. Healthcare utilization was measured as physician and dentist visits (dichotomous variables). Independent variables included demographic, immigration, health, and asset factors. Logistic regression was used to examine the impact of financial and real assets on healthcare utilization. Results: Asset ownership significantly enhances healthcare utilization among immigrants. Individuals with financial assets were 124% more likely to visit a physician and 66% more likely to visit a dentist, while real assets increased these probabilities by 35% and 22%, respectively. Sub-ethnic analyses showed that both asset types positively influenced physician visits for Asians and Europeans, but not Africans. For dentist visits, only Europeans showed significant associations with asset ownership, while Middle Easterners and Africans did not. Implications: These findings support the inclusion of asset ownership in the Andersen model, highlighting its relevance for immigrant populations. Asset-building programs could enhance healthcare utilization and improve immigrant health outcomes. Tailored policies are recommended to address the unique needs of diverse ethnic groups.

## 1. Introduction

In 2022, the number of immigrants in the United States reached a record 46.1 million, representing 13.8% of the total U.S. population [1]. This dramatic increase in the immigrant population has heightened concerns about their integration into American society, particularly given the socioeconomic disadvantages they often face as a result of migration [2,3]. These disadvantages typically manifest as a lower socioeconomic status, which can hinder successful integration and exacerbate immigrants’ vulnerability. These socioeconomic challenges often limit immigrants’ access to essential services, including healthcare, further compounding their difficulties in achieving full integration. Previous studies consistently highlight the inadequacy of healthcare access among immigrant populations, especially those with low socioeconomic status. Immigrants’ limited economic resources are closely related to their reduced use of health services, which ultimately leads to poorer health outcomes. As a result, there is a pressing need to understand the underlying factors that drive these disparities in healthcare access. Understanding the factors that influence immigrants’ health behaviors is crucial for improving their health outcomes [4].

Asset building refers to the accumulation of financial resources that can be utilized during periods of economic uncertainty [5]. Assets serve as a financial safety net when income becomes unstable. In the event of income loss, assets help sustain necessary expenses, including healthcare [5,6,7]. Households with assets are better equipped to continue using health services despite fluctuations in income. For immigrants, asset ownership may play an even more crucial role in healthcare utilization than income alone, as many immigrants face economic instability and downward mobility even after gaining permanent residency [8,9,10]. For those dealing with income insecurity due to factors like job instability or discrimination, assets can be vital for maintaining continuous healthcare access. Additionally, asset building fosters long-term financial security, which is essential for successful integration into American society and achieving upward mobility.

This study aims to apply Andersen’s Behavioral Model (1995) to examine the factors influencing healthcare utilization among immigrants, with a focus on asset ownership and cultural and ethnic differences. According to this model, health behavior is shaped by predisposing characteristics, enabling factors, and the need for care. This study introduces a novel perspective by expanding Andersen’s model to include asset ownership as an enabling factor, addressing a significant gap in the existing research on immigrant healthcare utilization. Asset ownership enhances healthcare access by providing financial stability, reducing out-of-pocket barriers, and enabling long-term planning for medical needs. For example, financial assets, such as savings, can be used to pay for routine checkups or urgent medical visits, while real assets, like homeownership, can provide collateral for health-related loans or expenses. These assets also act as a safety net, offering security against unforeseen health shocks.

This study specifically emphasizes enabling factors, expanding the model to include asset ownership as a critical component. In doing so, it examines how the economic conditions of immigrants, particularly their assets, affect their access to and use of health services. This approach provides a more nuanced understanding of the role economic stability plays in healthcare access for immigrant populations. By acting as buffers against economic shocks, assets indirectly support consistent access to health services, which is particularly critical for immigrant groups facing systemic barriers or low income levels. Additionally, this study offers insights into how cultural and socioeconomic factors intersect to shape healthcare utilization across different immigrant groups. This dual focus on asset building and ethnic differences offers unique insights into the economic and cultural dimensions of immigrant healthcare access, contributing to the development of more effective and tailored policies. By incorporating asset variables and examining ethnic differences, this study builds on previous research, contributing to the development of more effective policies and interventions for immigrant communities.

## 2. Theoretical Background

### 2.1. Andersen’s Behavioral Model

This study uses Andersen’s Behavioral Model of Health Service Use, originally developed in 1968 and refined in 1995, to understand health behaviors among immigrants. According to this model, health behavior is shaped by three main factors: predisposing characteristics, enabling resources, and the need for care. Predisposing characteristics encompass demographic factors (such as age, gender, and marital status), social structure (including education, ethnicity, employment, and family size), and health beliefs (such as values, attitudes, and knowledge about health) [11]. Enabling resources refer to various factors that support the use of health services, including personal resources (e.g., health insurance and income), family resources (e.g., regular sources of care and social support), and community resources (e.g., residence and region) [11,12]. Finally, the need for care is divided into perceived and evaluated needs: perceived need reflects an individual’s self-assessment of their health needs, while evaluated need is determined through a medical professional’s diagnosis. Through this framework, the behavioral model identifies the primary factors influencing health behaviors among immigrants.

### 2.2. Asset Holding

Sherraden (1991) posits that holding assets is one of the most important and powerful economic resources for future development [5]. Assets provide a range of welfare benefits that extend beyond immediate financial needs, significantly contributing to individuals’ and households’ long-term stability and well-being. According to Sherraden (1991), assets have several critical welfare effects, including (1) improving household stability; (2) creating an orientation toward the future; (3) stimulating the development of other assets; (4) enabling focus and specialization; (5) providing a foundation for risk-taking; (6) increasing personal efficacy; (7) increasing social influence; (8) increasing political participation; and (9) enhancing the welfare of offspring [5]. These welfare effects underscore assets’ critical role in helping households maintain emotionally and financially stable lives. When a household loses income-generating resources, assets can buffer the negative impacts of such a loss, ensuring that essential needs are met despite financial difficulties. In essence, asset holding suggests that households can utilize their accumulated assets as living resources in place of income during times of financial stress [5,7,13]. This buffering capacity highlights the importance of asset accumulation for long-term financial security and resilience.

Assets, therefore, are a significant factor in the well-being of immigrants. In the context of health behavior, assets could serve as a crucial enabling factor, similar to income, by providing the financial stability necessary to access healthcare services. Enabling factors, which include resources that facilitate access to care, are often considered more critical for vulnerable populations than other factors, such as needs or predisposing characteristics [12,13]. Thus, assets contribute to economic security and play a pivotal role in determining immigrants’ health behavior and service utilization.

## 3. Literature Review

### 3.1. Healthcare Utilization and Predisposing Factors

In Andersen’s health behavior model, predisposing factors are central in shaping individuals’ likelihood of using health services [11]. These factors, which include demographic characteristics, social structure, and health beliefs, collectively influence how individuals perceive and approach healthcare.

Demographic characteristics, such as age and gender, significantly affect healthcare utilization. For example, research highlights that females generally face higher health risks, including higher rates of chronic diseases, which lead them to use healthcare services more frequently than males [14,15,16,17]. This underscores how gender shapes both health risk levels and the tendency to seek medical care. Social structure, another important predisposing factor, encompasses elements like education, occupation, ethnicity, duration of stay in the U.S., and English proficiency. Education is closely linked to health outcomes, as lower educational attainment among immigrants correlates with higher poverty rates and less healthcare access [18,19]. Research shows that immigrants with higher education are more likely to visit physicians and have health insurance coverage [20]. Moreover, the length of time an immigrant has lived in the U.S. and their English proficiency affect health behaviors. Longer U.S. residency often correlates with increased healthcare utilization, although it may also be associated with adopting less healthy behaviors [21,22,23]. Limited English proficiency can hinder healthcare access and quality, as studies have revealed that higher proficiency is linked to more frequent doctor visits among Asians [10,24]. However, English proficiency may also correspond with certain unhealthy behaviors, such as increased smoking among females [25]. Overall, these predisposing factors significantly affect healthcare access and outcomes for immigrants, emphasizing the need for tailored healthcare strategies that address these challenges.

### 3.2. Healthcare Utilization and Enabling Factors

Enabling factors include resources and conditions that facilitate access to healthcare services. Traditional enabling factors in Andersen’s Behavioral Model are health insurance and income. However, evidence increasingly supports that assets, as financial buffers, should also be considered a critical enabling factor for healthcare access.

Health insurance is crucial in determining immigrants’ healthcare access. The absence of insurance presents a significant barrier, often delaying disease treatment and impeding preventive care [17,26,27,28]. The Migration Policy Institute (2004) reports that immigrants are over twice as likely as natives to be uninsured, which discourages preventive care and timely medical attention [28]. Income similarly affects healthcare access, with families of higher income typically accessing healthcare services more readily. However, research shows that immigrants generally have lower medical expenses compared to native-born individuals, despite often having worse health conditions [27]. For instance, immigrant children have 74% lower healthcare expenditures than native-born children, indicating a persistent healthcare access gap. Income disparities also influence dietary choices, with higher-income individuals, particularly women, being more likely to consume healthier foods [14].

### 3.3. Asset Holding as an Enabling Factor

Alongside insurance and income, assets should be considered a significant enabling factor within Andersen’s Behavioral Model. Research shows that assets positively impact health outcomes. For instance, Hahn (1993) found that homeownership benefits women’s health, while Joshi and Macran (1991) observed that asset holding correlates with lower mortality rates [29,30]. These findings suggest that assets provide financial stability, enabling individuals to access healthcare even without a consistent income. Economic empowerment interventions, such as the SUUBI program in Uganda, have demonstrated positive effects on health and mental health through asset ownership among vulnerable populations [31]. Additionally, Yadama and Sherraden (1996) argue that savings correlate with healthier behaviors like non-drinking, underscoring that assets offer security and promote healthier lifestyle choices. Thus, individuals with assets are more likely to engage in behaviors that foster long-term health [32].

### 3.4. Health Behavior and Need Factors

Need factors, which include both perceived and evaluated needs, are essential in understanding why and how individuals pursue healthcare. These factors account for subjective health perceptions and objective health evaluations that shape healthcare decisions and utilization.

Self-rated health, a prominent perceived need factor, is a strong predictor of future health outcomes as it captures an individual’s subjective health assessment. Research indicates that immigrants often rate their health more negatively than native-born individuals, which could reflect both perceived healthcare needs and barriers to accessing services [33].

Several studies link positive self-rated health with health-promoting behaviors. Among Latino immigrants, those with good to excellent self-rated health are more likely to engage in physical activity and other healthy habits [34]. Conversely, individuals who rate their health as poor may see a greater need for medical care or may be less motivated to maintain healthy behavior due to a lack of resources or a sense of hopelessness. Therefore, perceived need factors can drive people to either seek healthcare services or adopt healthier lifestyles, while evaluated need, based on medical assessments, determines the level and type of care required.

Based on a literature review, this study aims to shed light on the critical role of economic stability—particularly asset ownership—in facilitating healthcare access for immigrant populations. By examining financial and real assets as enabling factors, we can gain a deeper understanding of how economic resources influence healthcare behaviors across diverse immigrant groups. Furthermore, exploring ethnic differences in these associations will help identify unique healthcare access barriers and inform the development of tailored interventions that address the specific needs of each group. The findings from this study will ultimately contribute to more equitable healthcare access, empowering immigrant communities to achieve better health outcomes and supporting their successful integration into society.

## 4. Research Questions and Hypotheses

This study aims to examine whether asset holding among immigrants can serve as a facilitating factor that enhances their health behaviors. Specifically, it addresses the following research questions and tests the associated hypotheses:

Research Question 1: Is there an association between asset holding among immigrants and their healthcare utilization?

**Hypothesis** **1-1:** Financial asset holding is positively associated with an increased likelihood of visiting a physician or dentist among immigrants.

**Hypothesis** **1-2:** Real asset holding (e.g., property and home ownership) is positively associated with an increased likelihood of visiting a physician or dentist among immigrants.

Research Question 2: Are there ethnic differences in the association between asset holding and healthcare utilization among immigrants?

**Hypothesis** **2:** The association between asset holding and healthcare utilization varies across ethnic groups.

This study explores whether the impact of asset holding on healthcare utilization varies across different ethnic groups, highlighting potential disparities and the need for tailored interventions.

## 5. Methodology

### 5.1. Data and Sample

The data for this study comes from the National Immigrant Survey (NIS), which is designed to examine and evaluate various immigrant characteristics, including lifestyle, employment situation, economic status, and health condition. The dataset includes immigrant populations from various countries and utilizes a probability sampling method, making it nationally representative. The NIS comprises immigrants who acquired permanent residency status between May and November 2003. Baseline interviews were conducted with 12,500 adults aged 18 years or older, with 8573 completed interviews, representing a response rate of 68.6%. The dataset provides nationally representative data, which is essential for making broad and generalizable conclusions about the immigrant population in the United States. The NIS also includes detailed information on refugees, a critical immigrant population subgroup facing specific challenges. Moreover, the dataset contains comprehensive and specific variables related to asset holdings, allowing for a detailed analysis of how assets influence health behaviors among immigrants. These features make the NIS dataset particularly well suited for addressing the research questions in this study.

The unit of analysis in this study is individual adults. Of the 8573 adults surveyed, 4370 cases were selected based on specific inclusion criteria. First, 4187 cases (48.84%) were excluded because they did not respond to the questions on asset holdings. Since asset ownership is the key variable in this study, including cases with missing responses on this variable would have compromised the validity of the analysis. Asset ownership is central to the research questions and theoretical framework; thus, it was necessary to focus on cases with complete responses on this measure. Also, an additional 16 cases (0.19%) were excluded due to outliers or missing values for education and marital status variables. The multiple imputation method was applied to address missing values in the income variable, retaining 1350 cases (30.89% of the sample). Although these steps reduced the sample size, the results of this study can be generalized to all immigrants who hold legal permanent residency status in the U.S., as the sample includes information on both individuals with assets and those without assets. All statistical analyses were conducted using STATA 15.

### 5.2. Variables

#### 5.2.1. Dependent Variables

This study examined two dependent variables: physician visits and dentist visits. To measure physician visits, respondents were asked whether they had seen or spoken to a doctor in the previous 12 months. Responses were recorded as a dichotomous variable, with 1 indicating “yes” and 0 indicating “no”. For dentist visits, respondents were asked if they had seen a dentist in the previous 12 months. This variable was similarly coded as a dummy variable, with two categories: 1 for “yes” and 0 for “no”.

#### 5.2.2. Independent Variables

Two types of asset variables were used to measure asset holdings among immigrants. Due to a large number of missing values in variables that quantified the total amount of assets, this study utilized categorical asset-holding variables. These variables were based on yes or no responses to questions about whether the participants held various financial and real assets. The first independent variable, financial asset holding, was created by identifying whether respondents held any of the following: savings, certificates of deposit (CDs), treasury bills, stocks, Individual Retirement Accounts (IRAs), KEOGH plans, tax-favored accounts, or corporate/government bonds. If a respondent held at least one of these financial assets, they were classified as holding financial assets, making this a dichotomous variable: a score of 0 was given for not holding financial assets and 1 for holding financial assets. The second independent variable, real asset holding, was defined by ownership of real estate, a home, a business, or a farm. Respondents who owned at least one of these real assets were classified as holding real assets, and this variable was also measured as a dummy variable: a score of 0 was given for not owning real assets and 1 for owning real assets.

#### 5.2.3. Control Variables

Control variables in this study were divided into three categories: predisposing, enabling, and need factors. Predisposing variables include demographic and immigrant characteristics. Among the demographic characteristics, gender was measured as a dummy variable with the categories (1) male and (0) female. Age was calculated by subtracting the year of birth from 2004, and an age-squared variable was included to account for the assumption that the probability of visiting a doctor increases (or decreases) up to a certain age and then decreases (or increases) thereafter. Education was measured by the total years of schooling completed. Marital status was categorized into two groups, (1) married or cohabiting and (0) other, which included separated, divorced, widowed, and never married individuals. Employment status was similarly coded into two categories, (1) employed and (0) other, including unemployed, disabled, retired, and homemakers. The number of children was measured by counting the respondent’s biological children, while the number of household members was measured by counting the individuals living in the same household.

Immigrant characteristics included language proficiency, U.S. experience, visa admission category, and ethnicity. To measure English proficiency, a new dummy variable was created based on responses to questions about understanding and speaking English: individuals who understood or spoke English well or very well were coded as 1, while those who did not were coded as 0.

U.S. experience was calculated based on the formula from Akee and Yuksel’s (2008) study: 2004 minus the years of education in the U.S. minus the year the respondent left their country of origin [35]. The visa admission category was divided into four groups: (1) family preference, (2) employment preference, (3) refugee, and (4) diversity and other categories. For regression analysis, family preference was used as the base category, and three dummy variables were created for the other categories. Based on the respondent’s country of origin, an ethnicity variable was created with five categories: (1) Asian, (2) European, (3) Middle Eastern, (4) Latin American, and (5) African. For regression analysis, African was omitted, and four dummy variables were generated for the remaining categories.

Enabling factors included household earned income, which was calculated by summing the incomes from self-employment, wages, salaries, professional practice, and tips from both the respondent and their spouse. For regression analysis, household earned income was transformed into a proportional variable due to its skewed distribution. The health insurance variable included various sources, such as employer-provided insurance, private insurance, and insurance from a spouse. Health insurance was coded as a binary variable and scored as 1 if the respondent had insurance and 0 if they did not.

Need factors were measured in two ways. Self-rated health was assessed with the following question: “How would you rate your health: excellent (1), very good (2), good (3), fair (4), or poor (5)?” This study’s responses were reverse-coded, with poor = 1 and excellent = 5. Another need factor, doctor-diagnosed conditions, was assessed using questions about seven specific diseases: high blood pressure or hypertension, diabetes or high blood sugar, cancer or malignant tumor, chronic lung disease, heart disease, congestive heart failure, and stroke. If a respondent reported having at least one of these conditions, they were coded as 1 = yes.

### 5.3. Data Analysis

The data analysis for this study involved several key steps. Initially, descriptive analyses were conducted to summarize the characteristics of all variables, providing an overview of the dataset and helping to identify any patterns or trends within the data. The multiple imputation method was applied to handle missing values in the income variable. This approach allowed for the inclusion of cases with missing income data, thereby maximizing the sample size and reducing potential bias in the analysis. Finally, multivariate analyses were conducted to examine the research questions while controlling for all relevant variables. Logistic regression was used to predict the dichotomous dependent variables (physician and dentist visits) and to account for potential issues such as heteroskedasticity and unboundedness. Additionally, five separate logistic regression analyses were performed to explore ethnic differences in asset holding and healthcare utilization relationships. This thorough approach to data analysis ensured that the study’s findings were robust, addressing the key research questions while carefully considering the complexities of the data.

## 6. Results

### 6.1. Results of Descriptive Analyses

Table 1 provides a summary of the sample characteristics. Among adult immigrants, approximately half of the respondents were male (54.81%), and the other half were female (45.19%). One-third of the respondents (33.62%) were married. Regarding current employment status, nearly one-third of the respondents (27.59%) were employed at the time of the interview. The average age of the respondents was 38.70 years (SD = 0.17). On average, respondents had 1.59 biological children (SD = 0.03), and the average number of household members was 3.68 (SD = 0.03). The respondents had, on average, 13.44 years of education (SD = 0.07). This study considered immigration characteristics such as U.S. experience, language proficiency, visa admission category, and ethnicity. The results show that, on average, the respondents had 8.12 years of U.S. experience (SD = 0.12). In terms of English proficiency, 39.04% of respondents reported that they spoke and understood English very well or well. With respect to visa categories, the diversity category had the highest percentage of immigrants in the sample (35.26%), followed by family preference (30.85%), employment preference (25.65%), and the refugee category (8.24%). In terms of ethnicity, the largest group of immigrants came from Latin America and the Caribbean (38.12%), followed by Asia (28.49%), Europe, Australia, and Canada (21.69%). The smallest groups were immigrants from Africa (7.07%) and the Middle East and North Africa (4.62%). Regarding need factors, nearly one-third of respondents rated their health as excellent (38.18%), while only 0.80% reported poor health. Additionally, 87.44% of respondents indicated that a doctor diagnosed them with at least one disease. The average household income among immigrants was USD 42,326.04 (SD = 995.20), and 61.28% of respondents had health insurance. Concerning the independent variables, 58.95% of the sample held financial assets, and 37.48% held real assets. Finally, in terms of healthcare utilization, 60.38% of immigrants reported having visited a physician, while 53.49% had visited a dentist.

### 6.2. Results of Multivariate Analyses

Multiple logistic regression analyses were conducted to predict the impact of immigrants’ asset holdings on their likelihood of visiting both physicians and dentists. To estimate the independent effects of asset holdings, the analyses controlled for demographic, immigration, need, and enabling factors. The models for both physician and dentist visits were statistically significant (χ^2^ = 551.72 and χ^2^ = 461.47; *p* < 0.001).

As seen in Table 2, financial and real asset holdings, considered as enabling factors in Andersen’s Behavioral Model, were significantly associated with the likelihood of visiting both physicians and dentists. Asset holdings played a crucial role in accessing health services. Immigrants with financial assets were 2.24 times more likely to visit a physician and 1.62 times more likely to visit a dentist compared to those without financial assets, holding other variables constant (*p* < 0.001 for both). Additionally, owning real assets increased the odds of visiting a physician by 1.35 times (*p* < 0.001) and the odds of visiting a dentist by 1.22 times (*p* < 0.01). Health insurance also emerged as a significant factor in increasing healthcare utilization. The odds of visiting a physician for those with health insurance were 2.07 times greater than for those without insurance (*p* < 0.001), and similarly, the odds of visiting a dentist were 1.50 times greater for those with insurance (*p* < 0.001). However, household income, which is typically recognized as an enabling factor in Andersen’s Behavioral Model, was not statistically associated with visits to both physicians and dentists. These findings highlight the critical role of assets and health insurance in facilitating access to healthcare services.

Among the predisposing factors, the key demographic variables to note are years of education and ethnicity. Years of education were significantly associated with the likelihood of visiting both physicians and dentists. Each additional year of education increased the likelihood of visiting a physician by 1.03 times (*p* < 0.01) and a dentist by 1.04 times (*p* < 0.001). Regarding ethnicity, the odds of visiting a physician were 34% lower for Asians compared to Africans (*p* < 0.01). However, the odds of visiting a dentist were 1.62 times higher for Asians than for Africans (*p* < 0.01). Additionally, immigrants from Europe, Australia, and Canada had 3.18 times higher odds of visiting a dentist than those from Africa, while those from the Middle East and Latin America had 2.24- and 2.10-times higher odds, respectively, compared to immigrants from Africa (*p* < 0.001 for all).

Immigration characteristics, as predisposing factors, are crucial in understanding immigrants’ health behaviors. Immigration characteristics, including U.S. experience, ethnicity, and visa admission category, were significantly associated with physician and dentist visits. Each additional year of U.S. experience increased the probability of visiting a physician by 1.02 times (*p* < 0.001). Regarding the visa admission category, refugees had 51% lower odds of visiting a physician compared to those in the family preference category (*p* < 0.001). Similarly, refugees had 29% lower odds of visiting a dentist than those in the family preference category (*p* < 0.05). Conversely, immigrants in the employment and diversity visa categories had higher odds of visiting a dentist, with odds 1.33 times higher for those in the employment category (*p* < 0.001) and 1.21 times higher for those in the diversity category (*p* < 0.05), holding other variables constant.

In Andersen’s Behavioral Model, need factors such as current health and doctor-diagnosed conditions showed significant associations with physician visits. Holding other variables constant, a one-point increase in self-rated health decreased the odds of visiting a physician by 22% (*p* < 0.001). However, the odds of visiting a physician were 2.07 times greater for those with diagnosed conditions compared to those without any diagnosed conditions, holding other variables constant (*p* < 0.001).

Table 3 and Table 4 present the results of the logistic regression analyses for physician and dentist visits across different ethnicities. In the model for physician visits, both financial and real assets were significantly associated with physician visits for immigrants from Europe and Asia. For immigrants from the Middle East and Latin America, only financial assets showed a significant association with physician visits. However, neither financial nor real assets were significantly associated with physician visits for African immigrants. Additionally, having health insurance was a crucial factor in increasing physician visits for all ethnicities except for those from the Middle East.

As seen in Table 4, the model for dentist visits across different ethnic groups shows that asset holdings, both financial and real, were significantly associated with visiting dentists only for European immigrants. For immigrants from Asia and Latin America, only financial assets had a significant relationship with visiting a dentist. In contrast, there were no significant relationships between real asset holdings and visiting a dentist among immigrants from the Middle East, Asia, and Africa. However, income was the only significant factor for African immigrants in predicting dentist visits.

## 7. Discussion

The purpose of this study was to examine the relationship between asset holding and healthcare utilization among legal immigrants in the United States using Andersen’s Behavioral Model. Specifically, this study focused on determining whether asset holdings could serve as an enabling factor for healthcare utilization and explored potential ethnic differences in their impact. The findings of this study emphasize the role of economic stability, as measured through asset holdings, in facilitating access to healthcare services.

First, the findings demonstrate that asset ownership, both financial and real, is a significant enabling factor for accessing healthcare services, particularly physician and dentist visits. This finding aligns with previous research emphasizing the role of economic resources in healthcare access. For instance, Kawachi and Berkman (2020) demonstrated that financial assets provide a buffer against healthcare costs, facilitating better health outcomes [36]. Additionally, previous studies underscored that asset accumulation directly influences health-seeking behaviors, often outperforming income in predicting healthcare access [37,38]. These studies support the assertion that assets provide financial stability and enhance access to preventive care services.

Interestingly, while asset holdings were significant predictors, household income was not, which highlights the distinct role of assets. This is consistent with the findings obtained by West (2015), who argued that liquid assets, rather than income, are more directly tied to healthcare-related decision making [39]. This distinction underscores the importance of focusing on wealth distribution, rather than income alone, when addressing disparities in healthcare utilization.

Additionally, this study found that the impact of asset holdings varies across ethnic groups. For example, while financial and real asset holdings significantly influenced healthcare utilization among European and Asian immigrants, they were not significant predictors for African and Middle Eastern immigrants. Previous studies noted that racial and ethnic differences in asset ownership often reflect broader structural inequalities, which influence access to resources such as healthcare [39]. According to the Federal Reserve’s Survey of Consumer Finances (SCF), in 2022, nearly two-thirds of White families owned stocks compared to only 39% of Black families and 28% of Hispanic families [40]. Additionally, White families held these assets at much higher values. The median value of total stock holdings among White families in 2022 was USD 67,800, compared with USD 24,500 for Hispanic families and USD 16,500 for Black families [40]. These disparities suggest that European immigrants, who are more likely to have higher levels of asset ownership and greater financial resources, naturally benefit more from these assets when it comes to accessing healthcare services. The significant role of assets in healthcare utilization among European immigrants is thus consistent with their higher likelihood of owning valuable assets. In contrast, the lower levels of asset ownership among African and Hispanic immigrants may explain why asset holdings do not significantly influence their healthcare utilization. This underscores the broader issue of economic inequality across racial and ethnic groups, which has direct implications for access to healthcare and other essential services. This variation may also stem from differing cultural perceptions of healthcare. As shown by Braveman (2023), cultural and systemic barriers, including trust in the healthcare system and discrimination, disproportionately affect minority groups’ healthcare behaviors [41]. These factors may moderate the relationship between asset holdings and healthcare utilization, as observed in the current study.

English proficiency emerged as a critical factor in healthcare utilization, particularly among Asian and Hispanic immigrants. This finding aligns with earlier research by Rasi (2020), who highlighted language barriers as a significant obstacle to healthcare access among non-English-speaking immigrants [42]. Language proficiency facilitates navigation of the healthcare system, understanding medical information, and adherence to treatment plans, thus improving health outcomes.

## 8. Conclusions

This study highlights the critical role of economic stability, measured through asset holdings, in facilitating healthcare utilization among legal immigrants in the United States. The findings confirm that financial and real asset holdings serve as significant enabling factors for accessing healthcare services, such as physician and dentist visits. These results underscore the importance of wealth, rather than income alone, in predicting healthcare access and utilization.

Furthermore, the study demonstrates that the impact of asset holdings varies significantly across ethnic groups. While European and Asian immigrants benefit from asset ownership in healthcare access, African and Middle Eastern immigrants do not show similar patterns, reflecting broader structural inequalities in asset distribution. These disparities emphasize the need to address systemic barriers, such as economic inequality and cultural factors, to improve healthcare equity. Language proficiency also emerged as a crucial factor influencing healthcare utilization, particularly among Asian and Hispanic immigrants, reinforcing the importance of addressing linguistic barriers.

This study underscores several key policy implications. Efforts to reduce healthcare disparities should prioritize financial literacy programs and asset-building initiatives tailored to minority populations. Similarly, interventions that address language barriers, such as community health worker programs, can improve healthcare utilization and outcomes among immigrant populations.

This study has several limitations. First, the NIS data did not include undocumented immigrants, limiting the ability to compare healthcare utilization between undocumented immigrants and the native-born population. Second, because this study used cross-sectional data, it could not measure longitudinal asset accumulation over time. It is possible that healthcare utilization could be better explained by the process of asset accumulation rather than merely holding assets, suggesting an area for future research. Third, while the Andersen Behavioral Model provided a useful framework, it has inherent limitations in capturing the complex, interrelated influences of predisposing, enabling, and need factors on health behavior, which may not be fully represented in a single model or analysis. Additionally, the NIS data used in this study are somewhat dated, which may affect the relevance of the findings to current immigrant populations. However, the NIS remains a valuable data source due to its comprehensive coverage of legal immigrants, including detailed information on health, assets, and other critical variables that are not as readily available in more recent datasets.

Despite these limitations, this study reinforces the critical role of asset holdings as enabling factors in healthcare utilization while highlighting significant ethnic disparities. By situating these findings within the broader literature, the discussion demonstrates the complex interplay of economic, social, and cultural factors influencing healthcare access among immigrants. Addressing these disparities requires tailored policy interventions that consider both economic resources and systemic barriers, including language proficiency and discrimination. Such strategies are essential for improving healthcare equity and supporting the integration and well-being of immigrant communities in the United States.

## Figures and Tables

**Table 1:** The characteristics of all variables (N = 4370).

Variables		Mean (SD) or Frequency (%)	
Predisposing Factors Demographic Characteristics			
Age		38.70	(0.17)
Year of education		13.44	(0.07)
Number of children		1.59	(0.03)
Number of household members		3.68	(0.03)
Gender	Female	1975	(45.19)
	Male	2395	(54.81)
Marital status	Married	1469	33.62%
	Not married	2901	66.38%
Employment status	Working now	1205	27.59%
	Not working	3162	72.41%
Immigration Characteristics			
Years of U.S. experience		8.12	(0.12)
English proficiency	Very well/well	1706	39.04%
	Not well/not at all	2664	60.96%
Admission category	Family preference	1348	30.85%
	Employment preference	1121	25.65%
	Refugee	360	8.24%
	Diversity	1541	35.26%
Ethnicity	Asia	1245	28.49%
	Europe, Australia, and Canada	948	21.69%
	Middle East and North Africa	202	4.62%
	Latin America and the Caribbean	1666	38.12%
	Africa	309	7.07%
Need Factors			
Self-rated health	Excellent	1677	38.18%
	Very good	1322	30.28%
	Good	1051	24.07%
	Fair	291	6.67%
	Poor	35	0.80%
Doctor-diagnosed condition	Yes	3821	87.44%
	No	549	12.56%
Enabling Factors			
Household earned income		42,326.04	(995.20)
Health Insurance	Yes	2678	61.28%
	No	1692	38.72%
Financial asset holding	Yes	2732	62.52%
	No	1638	37.48%
Real asset holding	Yes	1794	41.05%
	No	2576	58.95%
Dependent Variables			
Physician visit	Yes	2635	60.38%
	No	1729	39.62%
Dentist visit	Yes	2336	53.49%
	No	2031	46.51%

**Table 2:** The results of logistic regression for healthcare utilization.

Variables	Physician Visit			Dentist Visit		
Predisposing Factors	B (SE)	Odds	95% CI	B (SE)	Odds	95% CI
Demographic characteristics						
Age	−0.02 (0.02)	0.98	[0.95, 1.02]	0.05 (0.02)	1.05 **	[1.01, 1.09]
Age (squared)	0.00 (0.00)	1.00	[1.00, 1.00]	0.00 (0.00)	1.00 **	[1.00, 1.00]
Years of education	0.03 (0.01)	1.03 **	[1.01, 1.05]	0.04 (0.01)	1.04 ***	[1.02, 1.06]
Gender (female)						
Male	0.30 (0.07)	1.35 ***	[1.21, 1.51]	0.35 (0.07)	1.42 ***	[1.26, 1.60]
Ethnicity (Africa)						
Asia	−0.41 (0.15)	0.66 **	[0.48, 0.90]	0.48 (0.15)	1.62 ***	[1.23, 2.14]
Europe, Australia, and Canada	−0.06 (0.15)	0.94	[0.71, 1.26]	1.16 (0.15)	3.18 ***	[2.47, 4.08]
Middle East and North Africa	0.06 (0.20)	1.06	[0.71, 1.58]	0.81 (0.20)	2.24 ***	[1.53, 3.27]
Latin America and the Caribbean	−0.18 (0.15)	0.83	[0.61, 1.12]	0.74 (0.15)	2.10 ***	[1.53, 2.88]
Marital status (not married)						
Married	−0.23 (0.08)	0.79 ***	[0.67, 0.93]	−0.10 (0.08)	0.91	[0.78, 1.07]
Number of children	−0.01 (0.03)	0.99	[0.93, 1.04]	−0.02 (0.03)	0.98	[0.93, 1.03]
Number of household members	0.02 (0.02)	1.02	[0.99, 1.06]	0.04 (0.02)	1.04	[1.00, 1.08]
Employment status (unemployed)						
Employed	0.03 (0.09)	1.04	[0.88, 1.22]	−0.02 (0.08)	0.98	[0.84, 1.14]
Immigration characteristics						
English proficiency (not well/not at all)						
Very well/well	−0.01 (0.08)	0.99	[0.85, 1.16]	0.28 (0.08)	1.32 ***	[1.15, 1.52]
Years of U.S. experience	0.02 (0.00)	0.02 ***	[1.01, 1.03]	0.00 (0.00)	1.00	[0.99, 1.01]
Visa admission (ref. family preference)						
Employment preference	−0.04 (0.11)	0.95	[0.79, 1.14]	0.28 (0.10)	1.33 ***	[1.13, 1.56]
Refugee	−0.71 (0.15)	0.49 ***	[0.37, 0.66]	−0.35 (0.14)	0.71 *	[0.53, 0.94]
Diversity/other	−0.05 (0.09)	0.95	[0.79, 1.15]	0.19 (0.09)	1.21 *	[1.02, 1.43]
Need Factors						
Self-rated health (good/fair/poor)						
Excellent/very good	−0.25 (0.04)	0.78 ***	[0.72, 0.84]	0.01 (0.04)	1.01	[0.93, 1.10]
Doctor diagnosed condition (no)						
Yes	0.59 (0.11)	1.80 ***	[1.47, 2.19]	0.12 (0.11)	1.13	[0.91, 1.39]
Enabling Factors						
Household earned income (logged)	0.03 (0.02)	1.03	[1.00, 1.06]	0.00 (0.02)	1.00	[0.96, 1.04]
Health insurance (no)						
Yes	0.73 (0.08)	2.07 ***	[1.73, 2.47]	0.40 (0.08)	1.50 ***	[1.27, 1.78]
Financial asset holdings (no)						
Yes	0.80 (0.08)	2.24 ***	[1.89, 2.66]	0.48 (0.08)	1.62 ***	[1.38, 1.90]
Real asset holdings (no)						
Yes	0.30 (0.07)	1.35 ***	[1.15, 1.58]	0.20 (0.07)	1.22 *	[1.02, 1.45]
Constant	−0.86 (0.48)			3.85 (0.48) ***		
LR X^2^ Pseudo R^2^	551.7 *** 0.09			461.47 *** 0.08		
Observations	4359			4362		

Note. *p* * < 0.05, *p* ** < 0.01, *p* *** < 0.001.

**Table 3:** The results of logistic regression for physician visits by ethnicity.

Variables	Asian		European		Middle Eastern		Latin American		African	
	Odds	95% CI	Odds	95% CI	Odds	95% CI	Odds	95% CI	Odds	95% CI
Predisposing Factors Demographic Characteristics										
Age	0.97	[0.79–1.19]	0.91 *	[0.74–1.12]	1.03	[0.84–1.26]	1	[0.82–1.22]	0.92	[0.75–1.13]
Age (squared)	1.00	[0.82–1.22]	1.00 *	[0.82–1.22]	1	[0.82–1.22]	1	[0.82–1.22]	1	[0.82–1.22]
Years of education	1	[0.82–1.22]	1.01	[0.83–1.23]	1.04	[0.86–1.27]	1.04 **	[0.86–1.27]	1.08	[0.89–1.33]
Gender (female)										
Male	1.05	[0.86–1.28]	1.52 **	[1.25–1.84]	1.63	[1.34–1.97]	1.55 ***	[1.28–1.88]	0.88	[0.72–1.08]
Marital status (not married)										
Married	0.97	[0.79–1.19]	0.80	[0.65–0.98]	0.79	[0.64–0.96]	0.79	[0.64–0.96]	0.66	[0.53–0.82]
Number of children	1.01	[0.83–1.24]	0.92	[0.75–1.13]	1.35	[1.11–1.63]	0.97	[0.79–1.19]	1.05	[0.86–1.28]
Number of household members	1.04	[0.86–1.27]	1.06	[0.88–1.30]	0.84	[0.69–1.02]	1.03	[0.84–1.26]	0.99	[0.81–1.21]
Employment status (unemployed)										
Employed	0.89	[0.72–1.09]	1.10	[0.89–1.35]	1.02	[0.83–1.25]	1.06	[0.86–1.29]	1.47	[1.20–1.79]
Immigration Characteristics										
English (not well/not at all)										
Very well/well	1.38	[1.12–1.71]	0.95	[0.77–1.17]	1.22	[0.99–1.51]	0.84	[0.68–1.04]	1.26	[1.02–1.56]
Years of U.S. experience	1.01	[0.83–1.24]	1.01	[0.83–1.24]	1	[0.82–1.22]	1.02 *	[0.84–1.25]	1.02	[0.84–1.25]
Admission (family preference)										
Employment preference	0.88	[0.72–1.09]	1.05	[0.86–1.29]	0.5	[0.41–0.61]	0.86	[0.70–1.07]	1.13	[0.92–1.39]
Refugee	0.55	[0.45–0.67]	0.34 ***	[0.28–0.42]	0.20*	[0.16–0.25]	0.46 **	[0.38–0.56]	1.00	[0.82–1.22]
Diversity/other	1.01	[0.83–1.24]	0.72	[0.59–0.88]	0.75	[0.62–0.92]	1.06	[0.86–1.29]	1.12	[0.91–1.38]
Need Factors										
Self-rated health (good/fair/poor)										
Excellent/very good	0.85 *	[0.69–1.05]	0.73 ***	[0.60–0.90]	0.54 **	[0.44–0.67]	0.8 ***	[0.66–0.97]	0.74	[0.61–0.91]
Doctor-diagnosed condition (no)										
Yes	1.33	[1.08–1.63]	2.40 ***	[1.95–2.94]	0.97	[0.79–1.19]	1.72 ***	[1.40–2.11]	3.80 **	[3.11–4.65]
Enabling Factors										
Household income (logged)	1.05	[0.86–1.27]	1.00	[0.82–1.22]	1.2	[0.98–1.47]	1.01	[0.83–1.23]	0.98	[0.80–1.19]
Health Insurance (no)										
Yes	2.41 ***	[1.96–2.97]	2.34 ***	[1.91–2.87]	1.37	[1.12–1.68]	1.73 ***	[1.41–2.13]	2.71 **	[2.21–3.33]
Financial asset holding (no)										
Yes	2.50 ***	[2.03–3.08]	2.74 ***	[2.23–3.36]	3.22 **	[2.62–3.95]	2.06 ***	[1.67–2.54]	1.63	[1.32–2.01]
Real asset holding (no)										
Yes	1.32 *	[0.89–1.95]	1.91 ***	[1.29–2.83]	1.04	[0.70–1.54]	1.21	[0.82–1.79]	1.34	[0.91–1.98]
LR X^2^	138.69 ***		214.16 ***		46.53 ***		162.45 ***		65.67 ***	
Pseudo R^2^	0.08		0.17		0.17		0.07		0.16	
Observations	1241		946		202		1661		309	

Note. *p* * < 0.05, *p* ** < 0.01, *p* *** < 0.001.

**Table 4:** The results of logistic regression for dentist visits by ethnicity.

Variables	Asian		European		Middle Easterner		Latin American		African	
Predisposing Factors Demographic characteristics	Odds	95% CI	Odds	95% CI	Odds	95% CI	Odds	95% CI	Odds	95% CI
Age	0.99	[0.67–1.47]	0.99	[0.67–1.47]	1.33 **	[0.90–1.97]	1.09 **	[0.74–1.61]	1.21	[0.82–1.79]
Age (squared)	1	[0.68–1.48]	1	[0.68–1.48]	0.1 **	[0.07–0.15]	1.00 *	[0.68–1.48]	1	[0.68–1.48]
Years of education	1.01	[0.68–1.49]	1.04	[0.70–1.54]	1.2**	[0.81–1.78]	1.07 ***	[0.72–1.58]	0.97	[0.66–1.44]
Gender (female)										
Male	1.51 **	[1.02–2.23]	1.33*	[0.90–1.97]	0.61	[0.41–0.90]	1.39 **	[0.94–2.06]	1.56	[1.05–2.31]
Marital status (not married)										
Married	0.81	[0.55–1.20]	0.89	[0.60–1.32]	0.43*	[0.29–0.64]	0.97	[0.66–1.44]	0.9	[0.61–1.33]
Number of children	0.96	[0.65–1.42]	1.12	[0.76–1.66]	1.64	[1.11–2.43]	0.92*	[0.62–1.36]	1.02	[0.69–1.51]
Number of household members	1.04	[0.70–1.54]	0.99	[0.67–1.47]	0.97	[0.66–1.44]	1.06	[0.72–1.57]	1.07	[0.72–1.58]
Employment status (unemployed)										
Employed	0.86	[0.58–1.27]	1.05	[0.71–1.55]	0.61	[0.41–0.90]	0.98	[0.66–1.45]	1	[0.68–1.48]
Immigration characteristics										
English (not well/not at all)										
Very well/well	1.68 **	[1.14–2.49]	1.2	[0.81–1.78]	0.82	[0.55–1.21]	1.37 *	[0.93–2.03]	0.97	[0.66–1.44]
Years of U.S. experience	1.00	[0.68–1.48]	1.01	[0.68–1.49]	0.99	[0.67–1.47]	1.00	[0.68–1.48]	1.02	[0.69–1.51]
Admission (family preference)										
Employment preference	1.44 *	[0.97–2.13]	1.38	[0.93–2.04]	0.25	[0.17–0.37]	1.59 *	[1.07–2.35]	1.65	[1.11–2.44]
Refugee	0.78	[0.53–1.15]	0.36 ***	[0.24–0.53]	0.22 *	[0.15–0.33]	0.79	[0.53–1.17]	1.43	[0.97–2.12]
Diversity/other	1.36	[0.92–2.01]	1.21	[0.82–1.79]	0.35	[0.24–0.52]	1.35*	[0.91–2.00]	0.79	[0.53–1.17]
Need Factors										
Self-rated health (good/fair/poor)										
Excellent/very good	1.08	[0.73–1.60]	0.83*	[0.56–1.23]	1.08	[0.73–1.60]	0.95	[0.64–1.41]	1.42 *	[0.96–2.10]
Doctor-diagnosed condition (no)										
Yes	0.9	[0.61–1.33]	1.6	[1.08–2.37]	0.6	[0.41–0.89]	1.05	[0.71–1.55]	2.08	[1.41–3.08]
Enabling Factors										
Household income (logged)	0.97	[0.66–1.44]	0.95	[0.64–1.41]	1	[0.68–1.48]	1.04	[0.70–1.54]	1.27 *	[0.86–1.88]
Health insurance (No)										
Yes	1.73 ***	[1.17–2.56]	1.13	[0.76–1.67]	2.73	[1.84–4.04]	1.44 **	[0.97–2.13]	2.16*	[1.46–3.20]
Financial asset holding (No)										
Yes	1.95 ***	[1.32–2.89]	1.71 **	[1.16–2.53]	1.65	[1.11–2.44]	1.47 ***	[0.99–2.18]	1.56	[1.05–2.31]
Real asset holding (No)										
Yes	1.18	[0.80–1.75]	2.14 ***	[1.45–3.17]	1.04	[0.70–1.54]	1.00	[0.68–1.48]	0.78	[0.53–1.15]
LR X^2^	107.66 ***		124.26 ***		50.69 ***		154.10 ***		59.36 ***	
Pseudo R^2^	0.06		0.10		0.18		0.07		0.16	
Observations	1241		945		202		1665		309	

Note. *p* * < 0.05, *p* ** < 0.01, *p* *** < 0.001.

## Data Availability

This study used secondary data from the NIS dataset, available at ICPSR. The data are anonymized and adhere to ethical guidelines.
